# 经皮冷冻猪肺的影像学和病理学研究

**DOI:** 10.3779/j.issn.1009-3419.2010.07.04

**Published:** 2010-07-20

**Authors:** 立志 牛, 静 王, 大卫 邱, 亮 周, 炳辉 吴, 刚 方, 俊 唐, 峰 穆, 海波 李, 保君 梅, 春梅 邓, 春娟 邓, 卓芳 郝, 克成 徐

**Affiliations:** 1 510300 广州，中国科学院广州生物院附属复大医院 Fuda Hospital, Affiliated to Chinese Academy of Sciences, Guangzhou 510300, China; 2 510300 广州，广州复大肿瘤医院 Fuda Cancer Hospital, Guangzhou 510300, China; 3 510300 广州，广州医学院第二附属医院 the Second Affiliated Hospital of Guangzhou Medical College, Guangzhou 510300, China

**Keywords:** 经皮冷冻, 肺冷冻, 冷冻治疗, Percutaneous cryosurgery, Cryoablation of lung, Cryotherapy

## Abstract

**背景与目的:**

肺癌已成为最常见死因的恶性肿瘤之一，对不能手术切除的肺癌，冷冻是一种安全可选择的消融治疗手段，但肺为含气组织，与冷冻肝脏、胰腺等实体器官不同，在理论上冷冻范围很难超过肿瘤边缘。本研究旨在通过正常猪肺模型实验了解不同冷冻-复温循环对肺部组织坏死范围的影响并探讨经皮冷冻肺治疗的技术方案。

**方法:**

采用6只平均体重为23 kg的正常西藏小型猪作为模型，在CT引导下选择猪肺上叶1点和下叶2点作为靶点，使用直径为1.7 mm的冷冻探针分别插入肺叶各靶点做经皮穿刺冷冻。左肺行冷冻10 min、复温5 min共2个周期的冷冻-复温循环；右肺先行冷冻5 min、复温5 min的2个冷冻-复温循环，然后行冷冻10 min、复温5 min的第3个冷冻-复温循环。左右肺的实验条件和实验方法均相同。实验中，观察CT影像下冰球的形态学变化。分别取冷冻后4 h、3 d和7 d的猪肺标本，观察其大体形态及其在光镜下的组织学变化。

**结果:**

猪肺冷冻过程中随着时间的延长和循环次数的增加，冰球逐渐增大；无论2个或3个冷冻-复温循环，所产生的冷冻范围（“假定坏死区”）在大体标本上均超过CT上冷冻过程中显示的冰球大小；冷冻后随着时间延长，组织学坏死区逐步增大，3天及以后，假定坏死区即为组织学坏死区。

**结论:**

经皮冷冻肺可以达到有效破坏靶组织的目的；在技术上，肺冷冻以3个冷冻-复温循环为佳；冷冻范围不强求冷冻“1 cm安全边缘”。上述研究结果对于简化冷冻治疗过程及减少并发症具有临床价值。

## Abstract (en)

**Background and objective:**

Lung cancer is one of the most commonly occurring malignancies and frequent causes of death in the world. Cryoablation is a safe and alternative treatment for unresectable lung cancer. Due to the lung being gas-containing organ and different from solid organs such as liver and pancreas, it is difficult to achieve the freezing range of beyond the tumor edge 1 cm safety border. The aim of this study is to examine the effect of different numbers of freeze cycles on the effectiveness of cryoablation on normal lung tissue and to create an operation guideline that gives the best effect.

**Methods:**

Six healthy Tibetan miniature pigs were given a CT scan and histological investigation after percutaneous cryosurgery. Cryoablation was performed as 2 cycles of 10 min of active freezing in the left lung; each freeze followed by a 5 min thaw. In the right lung, we performed the same 2 cycles of 5 min of freezing followed by 5 min of thawing. However, for the right lung, we included a third cycle of consisting of 10 min of freezing followed by 5 min of thawing. Three cryoprobes were inserted into the left lung and three cryoprobes in the right lung per animal, one in the upper and two in the lower lobe, so as to be well away from each other. Comparison under the same experimental condition was necessary. During the experiment, observations were made regarding the imaging change of ice-ball. The lungs were removed postoperatively at 3 intervals: 4 h, 3 d of postoperation and 7 d of postoperation, respectively, to view microscopic and pathological change.

**Results:**

The ice-ball grew gradually in relation to the increase in time, and the increase in number of cycles. The size of the cryolesion (hypothesis necrotic area) in specimens, over time, became larger in size than the size of the ice-ball during operation, regardless of whether 2 or 3 freeze-thaw cycles were performed. The area of necrosis was gradually increased over the course of time. The hypothesis necrotic area was equal to necrosis area 3 d after cryosurgery.

**Conclusion:**

Percutaneous cryoablation of the lung can achieve complete ablation of target tissue. The freezing technique may be different depending on the individual circumstances of each tumor. In technology, 3 freeze-thaw cycles are recommended, and the range of cryoablation's effective diameter may be not necessarily beyond the tumor edge at least 1 cm safe border during cryosurgery.

肺癌已成为最常见死因的恶性肿瘤之一，大部分确诊时已属中晚期，失去了手术切除机会^[1-4]^。对不能手术切除的肺癌，冷冻是一种安全可选择的消融治疗手段。研究^[5-7]^显示冷冻治疗肝癌、肾癌、胰腺癌等实体器官肿瘤时，一般认为应将冷冻范围超过肿瘤边缘，即所谓“1 cm安全边缘”，以达到全部消融靶组织的目的。但与冷冻肝脏、胰腺等实体器官不同，肺为含气组织，气体阻碍热量的传导，冷冻范围在理论上很难超过肿瘤边缘，从而难以获得完全消融。为了解决肺冷冻的技术问题，本研究观察了猪肺经皮冷冻的影像学、大体解剖学和组织学表现，试图在不同冷冻条件下探讨经皮冷冻肺的治疗技术方案。

## 1 材料与方法

### 1.1 实验动物

6只月龄均为12个月的西藏小型猪由南方医科大学动物实验中心提供。平均体重为23 kg，均有健康合格证书。常规喂养，实验前2周内接受常规检查，无饮食和行为异常。分别将6只猪标记为猪1、2、3、4、5和6。本研究设计获得复大医院伦理委员会的批准。

### 1.2 冷冻治疗系统

冷冻设备采用氩氦冷冻系统（Endorcare^TM^, CA, USA），该系统是根据焦耳定律设计，其原理是高压气体经过狭窄的喷嘴进入探针尖端，压力突然下降，不同的气体在局部产生不同的温度变化。氩气引起温度降低（可达-150 ℃），氦气可使温度升高（可达60 ℃）。

### 1.3 实验方法

对猪进行捆绑固定，将猪右侧卧位，对术区进行消毒、备皮等。用速眠新3 mL诱导麻醉，然后给予1.5%-2%浓度异氟醚维持全身麻醉，氧流量保持在1.5 L/min。在CT下，选择猪左肺上叶1点和下叶2点作为靶点，将1.7 mm冷冻探针分别插入左肺上叶和下叶各靶点。以100%的氩气激活探针，使针尖温度达（-140± 5）℃，持续10 min，改输氦气复温至（25±5）℃，持续5 min，为第1个循环。重复上述冷冻/复温循环，共行2个循环。左肺冷冻完成后，将猪左侧卧位，对猪的右肺冷冻与左肺冷冻时相似，同样选择3个靶点进行冷冻，不同点是做了3个循环的冷冻-复温循环：即先行2个冷冻5 min、复温5 min的循环，第3循环为冷冻10 min、复温5 min。术中对猪进行心肺监护。冷冻完毕后拔针，用明胶海绵止血。术后肌注速尿20 mg、地塞米松5 mg和青霉素160 IU。6只猪的实验条件和实验方法均相同。

### 1.4 术后观察

猪1在术后表现为体温降低，心跳减慢，于3 h40 min后死亡。其余猪麻醉苏醒后正常圈养，第1天即给予正常饮食，观察其生命体征和器官功能。喂养期间未给予静脉输液、抗生素或其它药物。

### 1.5 标本的选取

猪1死亡后立即解剖，猪2和3在术后3天、猪4、5和6在术后7天分别静脉给予戊巴比妥注射液100 mg/kg处死（此处置方法获得复大医院伦理委员会批准）。解剖取大体肺组织标本。将凡是黑色区域的冷冻部位假定为靶组织，称为“假定坏死区”。测量假定坏死区最大径数值，然后取中心区、边缘区及边缘外1 cm处的组织（图 1），福尔马林溶液固定、苏木精-伊红溶液染色后，在光镜下观察组织学变化。使用20倍的放大率，每个部位观察6个样本。

**1:**
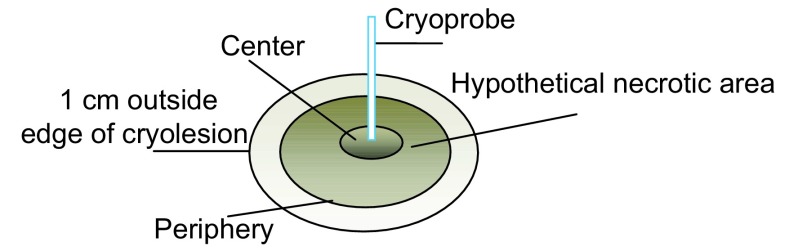
肺冷冻区标本选取示意图 Specimens selected draft of the freezing area of the lung

### 1.6 统计学分析

采用SPSS 13.0软件进行统计学分析，实验数值均使用Mean±SD描述，用配对样本*t*检验对计量资料进行分析，以*P* < 0.05为差异具有统计学意义。

## 2 结果

### 2.1 肺冷冻的CT下表现

CT下，冷冻区（冰球）表现为相对于肺的高密度实变影，冷冻探针表现为金属高密度影。最初形成的实变影相对模糊，与外周界限不清晰。随着冷冻时间的延长，实变影逐渐清晰，显示冰球逐渐增大。图 2显示猪3冷冻过程中CT显示的冰球演变，其大小随冷冻时间延长，冰球最大径增加由最初的2.4 cm→3.2 cm→2.9 cm→3.2 cm→3.5 cm→3.3 cm，呈现随着冷冻时间延长冰球逐渐增大的趋势。一般在复温后冰球范围较大，而再次冷冻后冰球变小，提示复温后渗出，引起冰球增大。

**2:**
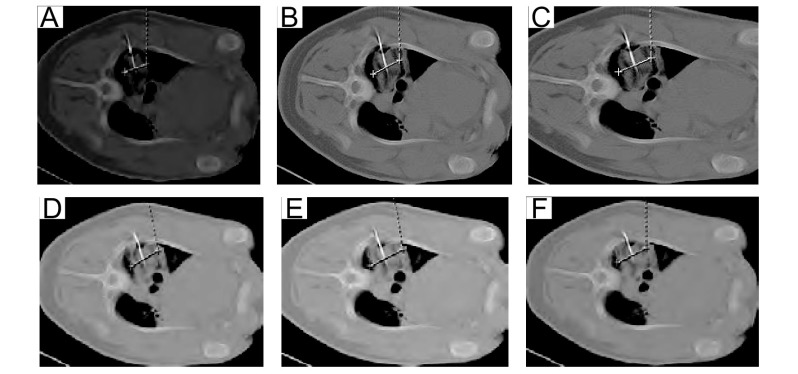
冷冻过程中（3个循环）冰球的CT表现 Sequential changes of the pulmonary parenchyma on computed tomographic scanning after freezing and thawing (3 cycles). A: 5 min after the beginning of first freezing; B: 5 min after the beginning of first thrawing; C: 5 min after the beginning of second freezing; D: 5 min after the beginning of second thrawing; E: 10 min after the beginning of third freezing; F: 5 min after the beginning of third thawing.

左肺行冷冻2个循环，冷冻总时间为20 min；右肺行冷冻3个循环，总冷冻时间也为20 min。右肺3个循环形成的冰球大于左肺2个循环形成的冰球（表 1）。图 3显示猪2不同冷冻-复温循环的左肺和右肺CT影像学变化。

**1:** 6只猪右肺与左肺的冰球和假定坏死区大小（cm） Compare hypothetical necrotic area with the ice-ball in both lungs (cm)

	*n*	Right lung	Left lung	*P*
Ice-ball	18	2.7±0.3	2.3±0.3	0.001
Hypothetical necrotic area	18	3.7±0.7	3.1±0.6	0.001

**3:**
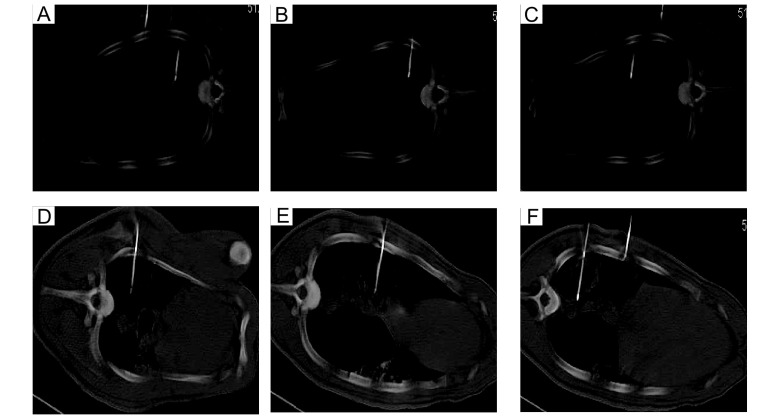
猪2不同冷冻-复温循环对CT上冰球大小的影响 Pig 2's ice-ball change under different freeze-thaw cycles. Above is left lung after 2 freeze-thaw cycles cryo, diameter was 1:2.1 cm (A), 2:2.1 cm (B), 3:2.4 cm (C), respectively; Below is right lung after 3 freeze-thaw cycles cryo, diameter was 4:2.6 cm (D), 5:2.7 cm (E), 6:2.6 cm (F), respectively.

### 2.2 肺冷冻的大体表现

猪1为冷冻后急性死亡，冷冻部位有出血，猪2、3和猪4、5、6分别在冷冻术后第3天和7天死亡，胸腔内有少量渗液和出血。6只猪肺的假定坏死区均呈黑色，与周围形成明显界限（图 4）。假定坏死区最大径猪1超过其它猪；6只猪右肺3个冷冻-复温循环的假定坏死区范围均超过左肺2个循环（表 1）。

**4:**
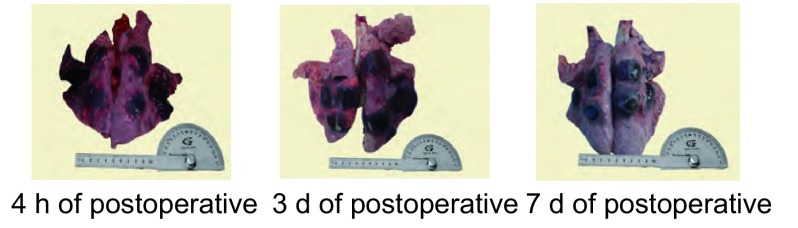
冷冻术后不同时间内双肺冷冻区的大体表现 The morphological description of both lungs'sample at different times after cryoablation

测定6只猪肺CT下冰球、假定坏死区最大径，结果如表 1。从表 1可见，CT影像下右肺的冰球大于左肺，右肺3个循环冷冻假定坏死区范围大于左肺2个循环，差异具有统计学意义（*P* < 0.05）。6只猪双肺冰球大小为（2.5 ±0.4）cm，假定坏死区大小为（3.4±0.7）cm，无论猪为急性死亡或术后3天、7天处死，双肺假定坏死区均较冰球大，差异具有统计学意义（*P* < 0.05）。

### 2.3 肺冷冻的组织学改变

6只猪肺的冷冻中心区在组织学上均呈现完全性坏死；在中心区周围的假定坏死区内，猪1有活存细胞存在，而其它猪肺呈现全部出血性坏死；在假定坏死区边缘外1 cm处，6只猪肺均无明显坏死（图 5）。此结果显示猪1的组织学坏死区小于假定坏死区，而冷冻3天后，其它猪肺假定坏死区和组织学坏死区大小一致。各猪左、右肺病变无明显差别。此外，急性死亡的猪1标本出血、水肿病变较其余两组严重，术后7天处死的猪4、5和6标本肉芽组织增生较其余两组明显。

**5:**
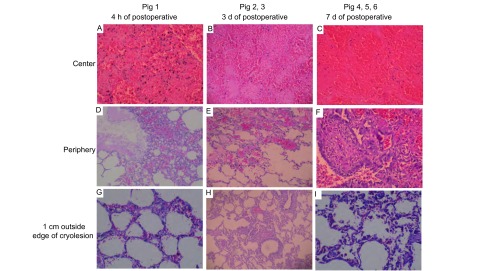
猪肺冷冻区光镜下的组织学改变(HE, ×20) Pathology of cryolesion of each pig under microscope (HE, ×20). A: lung tissues hemorrhage, necrosis; B: lung tissues hemorrhage, necrosis; C: lung tissues hemorrhage, necrosis; D: lung tissues vasodilatation, congest, lung tissues edema hemorrhage; E: alveolar septum congest edema, lung tissues hemorrhagic necrosis; F: necrosis around granulation tissue, alveolar epithelium and bronchiolar epithelium hyperplasia; G: alveolar structure is still maintained, part alveolar septum capillaries congests, few hemorrhage; H: alveolar structure is normal, part alveolar septum capillaries congests; I: alveolar structure is normal, surrounding alveolar capillaries congests, RBC and hemosiderin macrophages in part of alveolus.

## 3 讨论

冷冻疗法已愈来愈多用于实质器官如肝、肾和前列腺肿瘤的治疗。冷冻区内温度与距离冷冻探针的远近有关。中心部温度可能降至-160 ℃—-196 ℃，但在可见到的冰球外缘温度常为0 ℃。业已证明，冷冻温度为0 ℃—-30 ℃时，细胞无明显损伤；冷冻温度为-40 ℃—-50 ℃时，细胞死亡率明显增加。一般认为-40 ℃是引起靶细胞死亡的临界温度^[5, 8]^。在此温度以下的区域为有效冷冻区。为了达到这一目的，临床上沿用外科手术切除肿瘤的“1 cm安全边缘”概念^[9]^，即扩大冷冻范围，将有效冷冻范围扩展至肿瘤外至少1 cm^[6]^。

如同肝、肾的肿瘤冷冻一样，在理论上对肺癌的冷冻也应覆盖肿瘤周围10 mm宽的正常肺组织。但与肝、肾等实质器官不同，肺内含有气体，会阻碍低温传导，以致不能形成足够大的冰球，难以获得肿瘤周围“1 cm安全边缘”。为了克服这一障碍，有人主张增加冷冻-复温循环数。第1个循环冷冻复温后，肺泡内出血，将气体驱除，因此再次冷冻可使冰球扩大^[10]^。Izumi等^[11]^的实验显示，应用3 mm冷冻探针做第1个循环肺冷冻所形成的冷冻区为（3.3±1.4）cm^2^，而第2个循环后即增加至（10.6± 3.4）cm^2^（*P* < 0.05）。Kawamura等^[12]^主张做3个循环冷冻复温。他们发现用2 mm或3 mm冷冻探针冷冻可形成直径2.5 cm-3 cm冷冻区，而做3个循环冷冻-复温后冷冻区可扩展至4 cm。

我们应用正常猪模型在CT引导下做经皮肺冷冻实验，发现冰球大小随着冷冻-复温循环次数增加而增大，但我们也发现了文献中从未提及的一些现象：①肺冷冻，不管是2个循环抑或3个循环冷冻-复温，所产生的冷冻范围（假定坏死区）在大体标本上，均超过CT上冷冻过程中显示的冰球大小；②冷冻后近期，冷冻区边缘部有活存细胞，但3天后则边缘区均已坏死；冷冻后随着时间延长，组织学坏死区逐步增大，3天及以后，假定坏死区在组织学上全部呈现坏死，两者大小无明显区别，换句话说，假定坏死区就是组织学坏死区。

为何肺大体上冷冻区全部获得组织学坏死，而且范围超过CT上显示的冰球大小，尚难有合理解释，可能与肺泡组织对冷冻特别敏感、易于发生坏死或凋亡有关，但也可能由于冷冻后肺泡组织的血液供应受到严重破坏，进而引起次级或延迟性靶组织坏死，冷冻后随着时间延长坏死区扩大的事实可以此解释。

本研究说明，经皮冷冻肺可以达到有效破坏靶组织的目的。在技术上，以3个冷冻-复温循环为佳；冷冻范围不一定太大，不强求冷冻“1 cm安全边缘”，以免伤及肿瘤附近器官和组织，这在冷冻治疗临近心脏和大血管的肺肿瘤时尤为重要。此外，冷冻后7天，冷冻区可见胶原生成，肺泡上皮和支气管上皮的增生较明显，这也是冷冻区别于射频的优点，冷冻区可以保存组织支架，有利于恢复。

如果以上发现能进一步在临床上得到证实，则在肺癌冷冻治疗时，可能不一定需要把冷冻范围扩展到肿瘤外的“1 cm安全边缘”，肺冷冻范围不一定太大，只要稍微超过肿瘤本身范围即可，特别在CT上如果冰球超过肿瘤即足够，因为冷冻后范围会扩大，这对于减少正常组织破坏、简化治疗过程和减少并发症具有临床价值。我们应用经皮冷冻治疗中央型肺癌的经验显示，某些病人的肿瘤紧靠心脏或大血管，冷冻时无法获得“1 cm安全边缘”，但术后局部无复发，患者长期生存^[13]^，提示对肺癌的冷冻在技术上可能与冷冻其它实质性肿瘤不同。
